# Aberrant patterns of local and long-range functional connectivity densities in schizophrenia

**DOI:** 10.18632/oncotarget.18441

**Published:** 2017-06-12

**Authors:** Chuanxin Liu, Wei Zhang, Guangdong Chen, Hongjun Tian, Jie Li, Hongru Qu, Langlang Cheng, Jingjing Zhu, Chuanjun Zhuo

**Affiliations:** ^1^ Institute of Mental Health, Jining Medical University, Jining 272100, China; ^2^ Department of Psychiatry, Wenzhou Seventh People's Hospital, Wen Zhou 325000, China; ^3^ Department of Psychiatry, Tianjin Anding Hospital, Tianjin 300222, China; ^4^ Department of Psychiatry, Tianjin Anning Hospital, Tianjin 300300, China

**Keywords:** schizophrenia, magnetic resonance imaging, functional connectivity density

## Abstract

Schizophrenia is a disorder of brain dysconnectivity, and both the connection strength and connection number are disrupted in patients with schizophrenia. The functional connectivity density (FCD) can reflect alterations in the connection number. Alterations in the global FCD (gFCD) in schizophrenia were previously demonstrated; however, alterations in two other indices of the pathological characteristics of the brain, local FCD (lFCD) and long-range FCD (lrFCD), have not been revealed. To investigate lFCD and lrFCD alterations in patients with schizophrenia, 95 patients and 93 matched healthy controls were examined using structural and resting-state functional magnetic resonance imaging scanning. lFCD and lrFCD were measured using FCD mapping, and differences were identified using a two-sample *t*-test in a voxel-wise manner, with age and gender considered to increase variability. Multiple comparisons were performed using a false discovery rate method with a corrected threshold of *P*<0.05. Our analysis showed that lFCD was primarily decreased in the postcentral gyrus, right calcarine sulcus, and inferior occipital gyrus lobule, but increased in the bilateral subcortical regions. The differences in lFCD were more pronounced and complicated than those in lrFCD. In summary, in contrast with previous studies that focused on the connection strength, our findings, from the perspective of connection number, indicate that schizophrenia is a disorder of brain dysconnectivity, particularly affecting the local functional connectivity network, and support the hypothesis that schizophrenia is associated with a widespread cortical functional connectivity/activity deficit, with hyper- and/or hypo-connectivity/activity coexisting in some cortical or subcortical regions.

## INTRODUCTION

In the past two decades, several lines of evidence have converged to support that schizophrenia is a disorder associated with extensive disturbance of brain functional connectivity/activity [1–5]. Most of the previous studies suggest that hyper-connectivity/activity is mainly located in subcortical regions and some cortical-subcortical circuits. However, hypo-connectivity/activity is mainly located in the cortical regions, particularly in the cortical regions of the frontal and parietal cortex [6–12]. The frontal, temporal, parietal, and cingulate gyri, as well as the cuneus, precuneus, occipital gyrus, basal ganglia, hippocampus, and thalami are the crucial regions of alterations of functional connectivity/activity alterations in schizophrenia [6–12]. As early as 2011, Fornito et al. reviewed many previous studies and suggested that schizophrenia is associated with a widespread cortical functional connectivity deficit, and on this background, hyper- and/or hypo-connectivity was also observed in some cortical or subcortical regions [13]. Recently, a few studies based on graph theory have suggested that the brain network appears to have only a few well-localized regions (hubs) for the fast integration of neural processing, and their dysfunction could contribute to neuropsychiatric diseases, including schizophrenia [14–17]. However, most previous studies were focused on investigating alterations in the functional connection strength in schizophrenia [13, 18–22], and few studies investigated alterations in the functional connection numbers in schizophrenia [16, 23, 24]. Moreover, even fewer studies have investigated alterations in functional hubs in schizophrenia [25].

Functional connectivity density mapping (FCDM) can delineate brain network topology based on data from individual voxels [26–32]. It can determine the properties of the resting state networks that are associated with the major local functional connectivity density (lFCD) and long-range functional connectivity density (lrFCD) hubs in the cortical and subcortical brain regions [26–32]. In recent years, FCDM has been used in many studies to characterize aberrant functional connectivity hubs and investigate the aberrant FCD. These studies have yielded many pivotal findings. For example, Tomasi et al. found that lFCD hubs can be used as a biomarker for clinical applications in individuals with neuropsychiatric disorders [16] and that lrFCD hubs are more vulnerable to aging effects than short-range connections [30]. Additionally, Zhuo et al. found global FCD alterations in schizophrenia patients [24]. Shokri-Kojori et al. found that alcohol influences brain functional connectivity as well as its coupling with behavior [31]. In summary, multiple lines of evidence confirm that FCDM can be used to reveal abnormal functional activity hubs and abnormal functional connectivity densities in patients with mental disorders.

More importantly, in FCDM, the numbers of resting-state functional connections between a given voxel and other voxels in the brain are counted. This method is different from the traditional functional connectivity methods that focus on the functional connectivity strength (FCS) of two brain regions or voxels. FCDM focuses on the numbers of functional connections. FCS and FCD reflect different connectivity properties. FCS measures the connectivity strength between two voxels, regions or networks, and thus represents a one-to-one relationship. However, FCD reflects a one-to-many relationship. The FCD value increases as the number of functional connections between a particular voxel and other brain voxels increases, and a high FCD value suggests that this voxel plays a more important role in the information processing of the brain than voxels with low FCD values [26–33]. However, FCDM to has not previously been used investigate the alterations in lFCD, lrFCD and FCD hubs in schizophrenia patients.

In the present study, using FCDM methods, we aimed to delineate the functional hubs of patients with schizophrenia and to characterize the aberrant lFCD and lrFCD patterns in these individuals. We hypothesized that the lFCD and lrFCD would be altered in schizophrenia patients and that the space distribution of aberrant lFCD or lrFCD and the aberrant pattern (increased or decreased) of lFCD or lrFCD would be different in schizophrenia patients from those in healthy individuals.

## RESULTS

### Demographic and clinical characteristics

A total of 95 patients and 93 healthy controls were enrolled in the study. There were no significant differences in gender (*χ^2^*=1.35, *P*=0.25) and age (*t*=0.48, *P*=0.63) between the two groups (Table 1). Among the 95 patients, 87 received medication during the MRI examinations. The duration of illness and antipsychotic dosage (chlorpromazine equivalents) were 121.42±92.80 months and 446.54±341.60 mg/d, respectively. The PANSS scores of the positive subscale, -negative subscale, and general psychopathology subscale were 17.10±7.92, 20.30±9.13, and 34.14±10.83, respectively (Table 1).

**Table 1:** Demographic and clinical characteristics of study groups

Characteristics	Schizophrenia patients	Healthy controls	*t*	*P*
Number of subjects	95	93		-
Age (years)	33.6 ±7.8	33.0±10.2	0.482	0.633
Gender (female/male), *n*	41/54	48/45	1.654	0.246
Antipsychotic dosage (mg/d), chlorpromazine equivalents	446.54±341.60		-	-
Duration of illness (months)	121.42±92.80			-
PANSS Positive score Negative score General psychopathology Score Total score	17.10±7.92 20.30±9.13 34.14±10.83 71.5±23.20		- - - - -	-

PANSS, Positive and Negative Syndrome Scale. Data are shown as the means ± standard deviation.

### Distributed specificity of FCD hubs in patients and healthy controls

**Figure 1:**
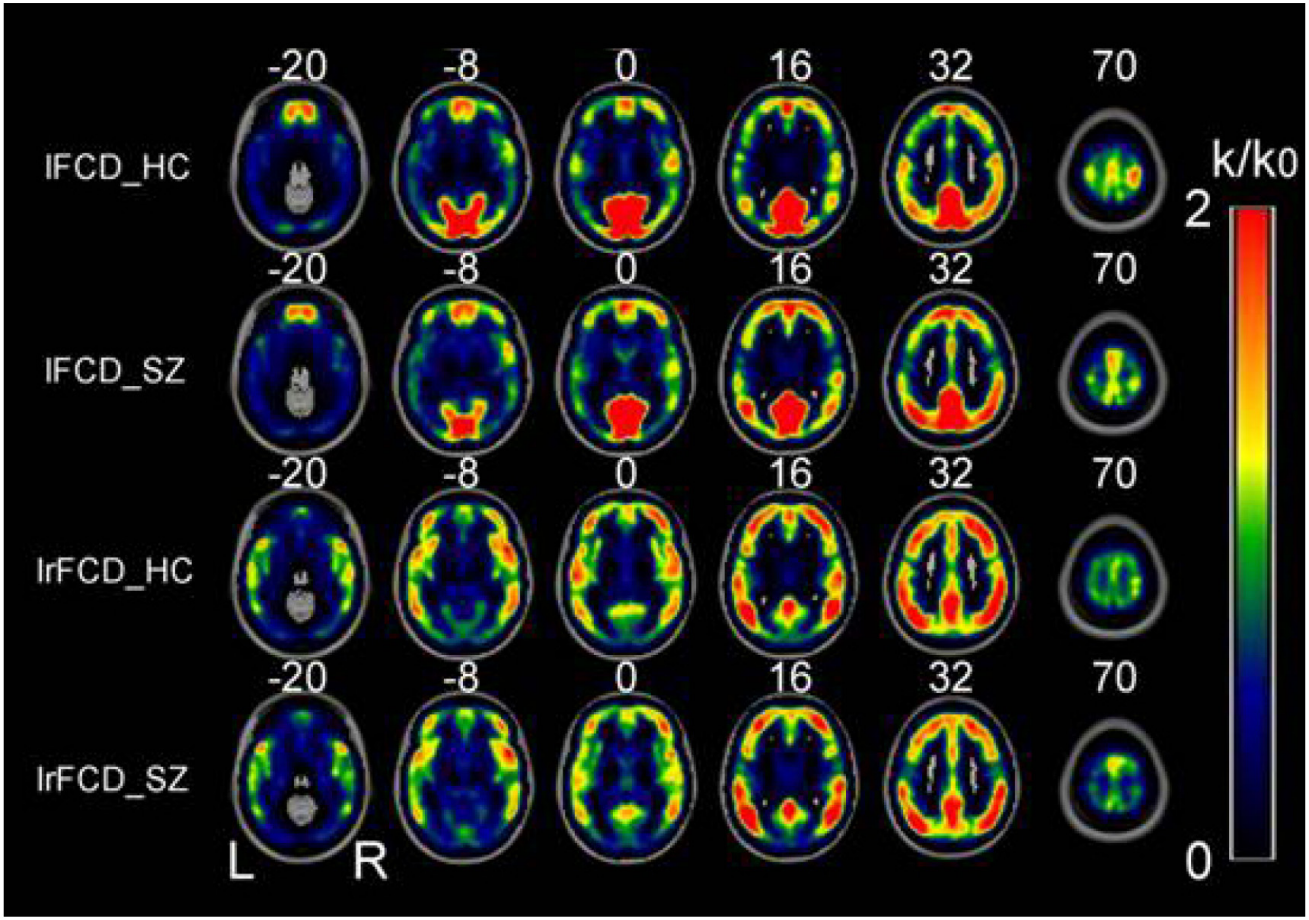
Spatial distributions of the average rescaled lFCD and lrFCD hubs in healthy control individuals and schizophrenia patients FCD: functional connectivity density; HC: healthy controls; lFCD: local FCD; lrFCD: long-range FCD; SZ: schizophrenia patients; L: left; R: right.

In patients and healthy controls, the hubs with the highest FCD were consistently located in the precuneus and the inferior parietal lobe, followed by the superior temporal gyrus, medial prefrontal cortex, and dorsal lateral prefrontal cortex. The FCD hubs were also consistently distributed in similar brain regions in the patients and the healthy controls. Most of these FCD hubs were components of the default mode network and sensory cortices (Figure 1). The distribution of the FCD hubs was generally consistent with the findings of Tomasi et al. [32]. The hub distribution did not differ between the two groups.

### FCD changes in schizophrenia patients

**Figure 2:**
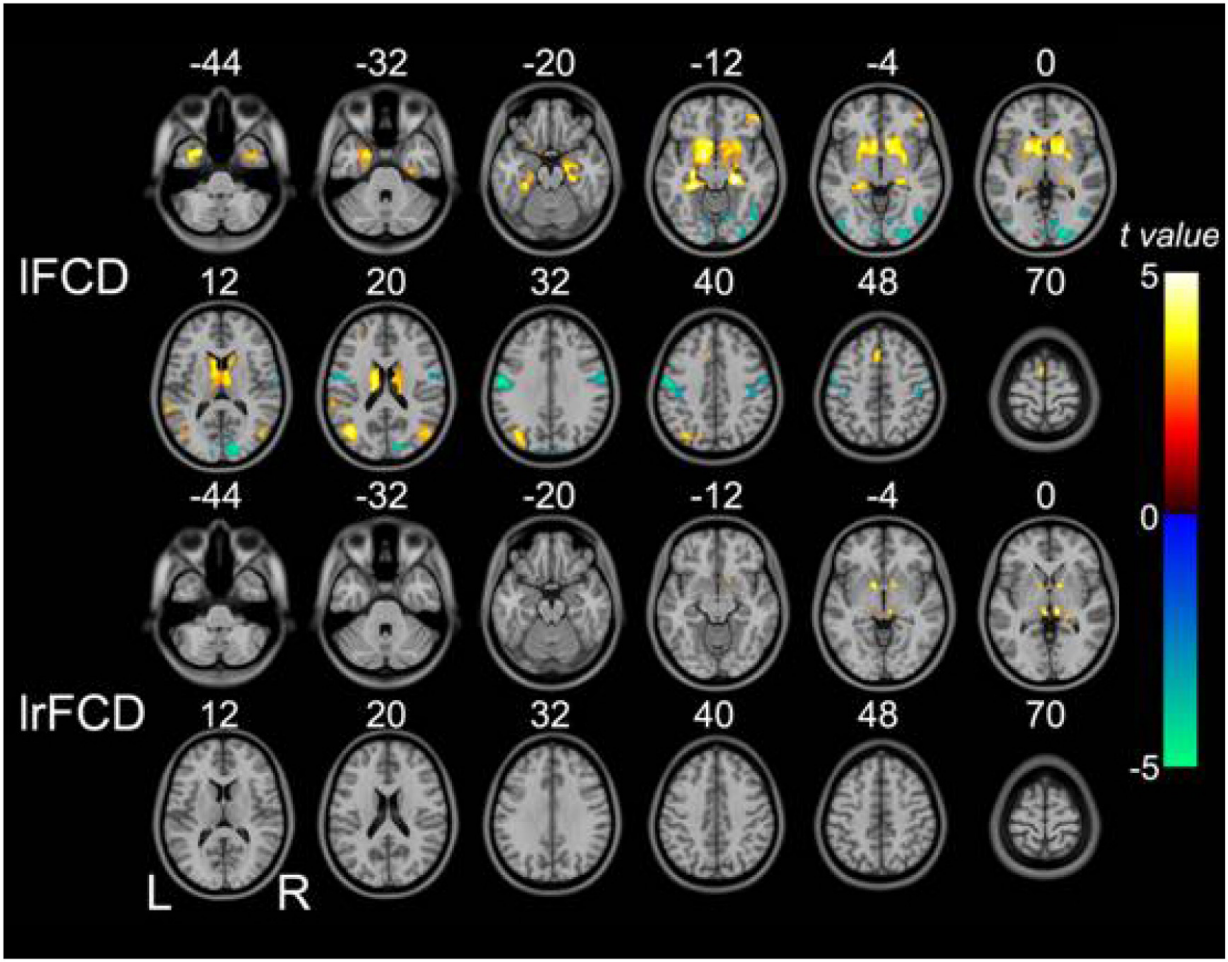
Brain regions in which lFCD and lrFCD differed significantly between schizophrenia patients and healthy controls Voxel-based analysis shows brain regions with significant group differences in lFCD and lrFCD (*P*<0.05, false discovery rate corrected). Warm colors represent significantly increased FCD in schizophrenia patients. Cold colors denote significantly decreased FCD in schizophrenia patients. FCD: functional connectivity density; lFCD: local FCD; lrFCD: long-range FCD; L: left; R: right.

Voxel-wise analysis showed decreased lFCD primarily in the bilateral postcentral gyri, inferior temporo-occipital conjunction (TOC), and right calcarine sulcus. lFCD was increased in the bilateral subcortical regions (*i.e*., the bilateral putamen, caudate, pallidum, and thalami), hippocampus/parahippocampus complex, temporal pole, middle TOC, supplementary motor area, right orbital frontal gyrus, and left superior temporal gyrus. We found increased lrFCD only in the bilateral thalami and pallidum (Figure 2).

### Associations between FCD and clinical variables

No associations were observed between the lFCD and lrFCD values and the antipsychotic dosage, illness duration, or any PANSS score.

## DISCUSSION

In this study, we found that both lFCD and lrFCD were altered in schizophrenia patients. The brain regions with aberrant lFCD were more diffuse and larger than the brain regions with aberrant lrFCD. The altered patterns of lFCD were also more complex than lrFCD, with decreased and increased lFCD both observed in schizophrenia patients compared with only one inter-regional increase in lrFCD. These findings suggest that the disturbance in local functional connection numbers was pronounced in schizophrenia patients, which is partially in agreement with a previous observation that lFCD was decreased in the primary sensorimotor area and increased in frontal areas [23]. Although previous findings for some brain areas with aberrant lFCD did not agree with our findings, this previous study supported our findings to some extent. Contrary to lFCD alterations and lrFCD alterations, the distributions of both lFCD and lrFCD hubs were not significantly different between the patients and healthy controls. This phenomenon is not unusual. Tomasi et al. also found similar distributions of FCD hubs between children with attention-deficit/hyperactivity disorder and healthy controls [32]. Overall, our findings and Chen et al.'s findings [23] converge to support the hypothesis that schizophrenia is a disorder of brain dysfunction that particularly affects the local functional connectivity network [3, 13, 14, 17, 34, 35].

Fornito et al. suggested that schizophrenia is associated with a widespread cortical functional connectivity deficit. On this basis, hyper- and/or hypo-connectivity have also been observed in some cortical or subcortical regions [13]. In the present study, we found that decreased lFCD was primarily located in the posterior cortices, whereas increased lFCD and lrFCD were primarily located in the subcortical and limbic system regions. Under this background, some scattered increased or decreased lFCD areas were distributed in different cortical regions. A considerable number of studies that adopted other methods also found that subcortical functional activity alterations usually occur in an increased pattern, whereas local cortical functional activity alterations usually manifest as a pattern of hypo- and hyper-functional activity.. For example, Turner et al. reported that the amplitude of low-frequency fluctuations (ALFF) was significantly increased in the putamen, amygdala, hippocampus, and hypothalamus [8]. Yu et al. [7] also described decreased regional homogeneity (ReHo) in the precentral gyrus and the middle occipital gyrus, but increased ReHo in the medial prefrontal cortex and the anterior insula. Ren et al. [9] reported that ALFF was decreased in the right inferior and left superior frontal gyrus, bilateral medial frontal gyrus, bilateral inferior parietal lobule, and precuneus, but ALFF was increased in the bilateral occipital regions. Consistent with the above-mentioned studies, our findings support Fornito's hypothesis from the perspective of connection numbers [13].

In our analysis, the lFCD and lrFCD values also showed no statistical correlation with the antipsychotic dosage. This is consistent with our previous reports that the global FCD value was not correlated with antipsychotic dosage. Previous studies also reported that there was no relationship of illness duration, symptom severity, and antipsychotic dosage with brain functional connectivity [9, 32, 36]. These studies and our findings indicate that BOLD signal alterations may track the presence of psychosis, instead of its severity or duration [9], and are not linearly correlated with the antipsychotic dosage [32, 36].

Although we found aberrant functional connection numbers in schizophrenia patients, the significance of our research is limited. Our study was a cross-sectional study, and the findings of this work reflect only the cross-sectional brain imaging characteristics of schizophrenia. Regarding the possible confounding effects of the medications the patients had received, we cannot be absolutely sure that the changes in lFCD and lrFCD observed in schizophrenia patients were due to the disease itself rather than the medication. A large-sample, extended follow-up study enrolling first-episode, drug naïve patients would enable a dynamic characterization of the developmental trajectory of the brain imaging characteristics of schizophrenia. These findings could be associated with the pathogenesis of the illness, its duration, and treatment interventions. Based on brain imaging results and the observed developmental trajectory of the imaging characteristics of schizophrenia, treatment targets could be explored, and treatment efficacy predictors based on pretreatment MRI data could be established. Such efforts could greatly improve the early cure rate; however, this goal will require the cooperation of many study centers.

## MATERIALS AND METHODS

### Study population

This study was conducted from February 2013 to November 2014 and was approved by the Ethics Committee of Tianjin Anning Hospital (No. 2012-H-008). All participants provided written informed consent. Two hundred right-handed participants, including 98 schizophrenia patients and 102 healthy controls, were enrolled in the present study. Patient diagnoses and the durations of illness were defined with the consensus of two professional psychiatrists who used the Structured Interview for DSM-IV (SCID) Axis I Disorder criteria. The lifetime absence of psychiatric illnesses in the healthy controls was confirmed by using the non-patient edition of the SCID (SCID-NP). Moreover, control subjects were interviewed to exclude those with a known family history of mental disorders in first-degree relatives. The exclusion criteria included 1) previous head trauma with a consciousness disturbance lasting more than 5 minutes; 2) a history of drug or other substance abuse; 3) pregnancy; 4) organic illness, including cardiovascular disease; or 5) a neurological disorder, as diagnosed by an interview and medical record review. The image quality was assessed by a professional radiologist. Consequently, three patients with schizophrenia and nine healthy controls were excluded due to poor image quality. Therefore, 95 patients and 93 healthy controls were included for further analysis. For all participants, the Positive and Negative Syndrome Scale (PANSS) was used to quantify the clinical symptoms of psychosis.

### Magnetic resonance imaging (MRI) data acquisition

MRI was performed using a 3.0-Tesla MR system (Discovery MR750, General Electric, Milwaukee, WI, USA). Fitted foam padding and earplugs were used to minimize head motion and reduce scanner noise, respectively. Sagittal 3D T1-weighted images were acquired using a previously described brain volume (BRAVO) sequence [37]. Then, resting-state functional magnetic resonance imaging (fMRI) was performed using a gradient-echo single-short echo planar imaging sequence with previously described parameters [37]. All participants were instructed to keep their eyes closed, to stay relaxed, to minimize movement, to think of nothing in particular, and to stay awake during the fMRI scans.

### fMRI data preprocessing

Preprocessing of resting-state fMRI data was conducted using SPM8 (http://www.fil.ion.ucl.ac.uk/spm). The first 10 volumes for each participant were discarded to avoid the use of data collected before participants could adapt to the scanning noise and the signal could reach equilibrium. The remaining volumes were corrected for the acquisition time delay between slices, and then, realignment for motion between time points was applied. The fMRI data of all participants were within defined motion thresholds (*i.e*., translational or rotational motion measures <2 mm or <2°). Frame-wise displacement (FD), which reflects the volume-to-volume changes in head position, was also calculated. Several nuisance variances, including the estimated six motion parameters, average blood oxygen level dependent (BOLD) signals of the ventricular and white matter, and the first-time derivatives, were removed through a linear regression analysis. The head motion-induced signal spike has been reported to significantly contaminate the final resting-state fMRI data, even after corrected for the six motion parameters via regression [25]. For this reason, we performed regression for the spike volumes if the FD of the specific volume was >0.5. Band-pass filtering of the data was applied within the frequency range of 0.01–0.08 Hz. For normalization, individual structural images were linearly co-registered with the mean functional image first and then to the MNI space. Finally, the filtered functional volumes underwent spatial normalization to the MNI space using the co-registration parameters, and then resampling into 3-mm cubic voxels was performed.

### FCD calculation

A program written in-house on the Linux platform was used for calculating the FCD of each voxel, as previously described by Tomasi and Volkow [26–32]. The FCD was calculated only forthe cerebral gray matter mask regions. For FCDM, the lFCD at x_0_ was computed as the number of elements in the local functional connectivity cluster, k(x_0_). The calculated then proceeded through all values of x_0_ for all qualified voxels in the brain. The strength of the lrFCD was defined as the global FCD [28] minus the lFCD. Grand mean scaling of lFCD and lrFCD was achieved by division by the mean value for all brain voxels to better normalize the distribution. Finally, the FCD maps were spatially smoothed using a 6×6×6-mm^3^ Gaussian kernel to minimize inter-subject differences in functional brain anatomy. As recommended by the developer of FCDM, Pearson's linear correlation was used to evaluate the strength of the functional connectivity between voxels. A correlation coefficient of r>0.6 for two voxels indicated a significant connection between them. This threshold and the above-mentioned in-house script have been confirmed to be optimal for calculating FCD [25–31]. Therefore, in the present study, we used the threshold of r=0.6 to calculate FCD.

### Statistical analysis

Demographic and clinical data were analyzed for statistical significance using SPSS version 19.0 software (SPSS, Chicago, IL, USA). Differences in dosages of antipsychotic agent and disease durations between the two groups were determined by two-sample *t*-tests, and the significance of the difference in gender between the two groups was determined by the chi-square test. Differences in lFCD and LrFCD were compared using two-sample *t*-tests, in a voxel-wise manner, with age and gender considered nuisance variables. A false discovery rate method with a corrected threshold of *P*<0.05 was applied for multiple comparisons.

## CONCLUSION

This study found both aberrant lFCD and lrFCD in schizophrenia patients, and the variation in lFCD was more pronounced and more complicated than that of lrFCD. The lFCD and lrFCD values also showed no statistical correlation with the antipsychotic dosage, illness duration, or severity of the illness. Simultaneously, we found that the distributions of lFCD and lrFCD hubs did not differ significantly between the patient and control groups. Our findings support the hypothesis that schizophrenia is a disorder of brain dysconnectivity that particularly affects the local functional connectivity network, which is characterized by coexisting hyper- and hypo-connectivity in some cortical or subcortical regions.
